# Locating disease spread: cholera to coronavirus and the art of the image

**DOI:** 10.1098/rsfs.2021.0014

**Published:** 2021-10-12

**Authors:** Katy Barrett, Geoffrey Belknap

**Affiliations:** Curatorial, Science Museum Group, Science Museum, Exhibition Road, London SW7 2DD, UK

**Keywords:** coronavirus, image-making, medical illustration, objectivity

## Abstract

This article considers the history of medical image-making to shed light on an aspect of the COVID-19 pandemic. Starting from a contemporary art commission in the Science Museum's ‘Medicine: The Wellcome Galleries’, we look at the role of image production and presentation in understanding the spread of disease. From the intertwined histories of art and scientific image-making, we explore five examples of iconic medical images, by John Snow, Florence Nightingale, Arthur Schuster, Donald Caspar and Aaron Klug, ending with a model of the coronavirus by the Cambridge University Laboratory of Molecular Biology. We trace how images have provided the means for discovery, for description and for diagnosis and outline the different ways in which diseases have been located in the history of the medical image: in the community, in the body, in the cell and on the image itself.

## 1. Introduction

The production and presentation of scientific ideas involves the making of aesthetic choices. In the twentieth and twenty-first centuries, we have become used to the idea that science is objective, presented to us unmediated by the verbal and visual tools used to develop and communicate scientific findings [1]. Yet, the coronavirus pandemic has brought home, perhaps more than any other scientific event, how data only result in action if they are read/seen and understood. The graphs, illustrations and visuals shown to us regularly by governments across the world have been crucial in persuading the public of the spread of cases and the successful impact of restrictions put in place as a curb. Images have power.

**Figure 1.**
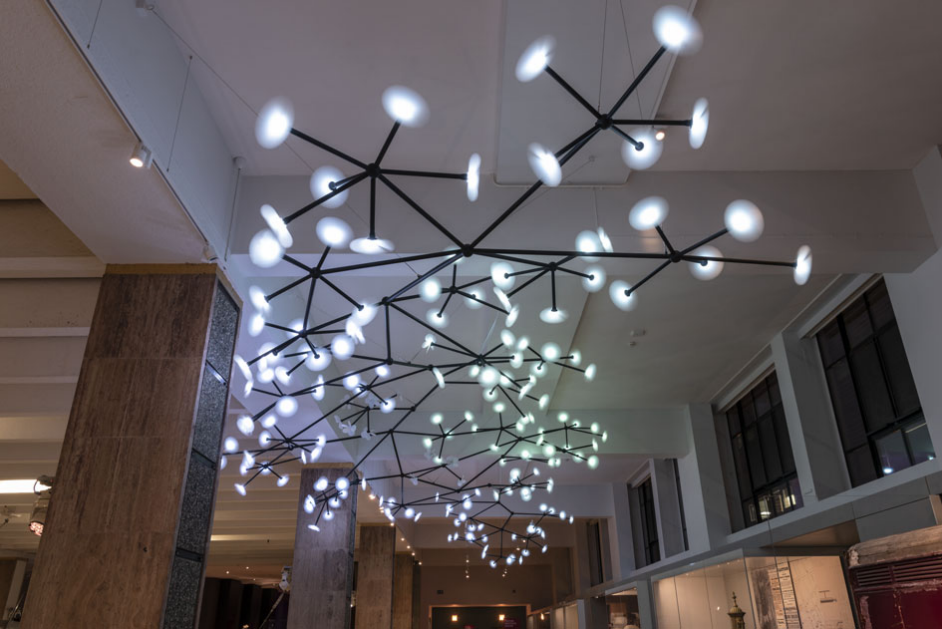
Bloom installed in ‘Medicine: The Wellcome Galleries’ at the Science Museum, London. Science Museum Group © The Board of Trustees of the Science Museum.

In *Medicine: The Wellcome Galleries* at the Science Museum, London, one ‘image’ has had particular power over the course of the pandemic. *Bloom* by Danish designers Studio Roso (founded 2008) is a three-dimensional kinetic artwork, installed hanging from the ceiling of the ‘Medicine and Communities' gallery. Designed to evoke the spread of disease, it resembles a large network diagram, with a pattern of black steel rods connecting nodal points and branches that end in translucent propellers. These propellers spin and light-up in a series of different colours and patterns that create narratives of disease spread. The colours have different intensity and tone, the propellers spin at different speeds, light and movement spreads both fast and slowly across the sculpture. Each propeller might be a person, a family, a community, a city or a nation. As propellers come to a sudden halt and the light goes out; has this person recovered from the disease, or sadly died? (figure 1).

When conceived and installed in the galleries in 2019, *Bloom* was intended both to give viewers a visual means to understand the spread of an epidemic or pandemic disease, and to respond to the history of illustration and use of diagrams within science and medicine. It is a site-specific installation, conceived for a particular location and context, within the public history of medicine, but connected to myriad others. Among many examples, it was inspired by network diagrams produced by the ‘Embedding the Human Disease Network’ at Stanford University.^1^ Discussing their commission for this artwork in 2020, Studio Roso commented that, ‘being visual people, we were intrigued by the diagrams and maps of disease transmission networks and epidemiological diagrams that experts use to predict the paths that epidemics will follow across the globe … These networks are an important part of epidemiological studies and are also visually very beautiful’ [2]. *Bloom*, in its function and conception, thus captures the dual roles of such scientific images, as both tools for experts in producing their work, and as vehicles for communication. It also locates this image in a particular context. All of these require aesthetic form, a choice of style, or to be ‘very beautiful’, as the artists put it.

With this poignantly relevant object from the collections of the Science Museum Group as our starting point, this article considers the history and significance of image-making practices in how the disease has been visualized and located. It is not intended to provide a comprehensive historical narrative, but rather to consider a series of case studies from the nineteenth to twenty-first centuries that highlight how images have been used to understand the disease, and what we can learn from these examples in the present moment. Working through different methods of observation, and different techniques of representation, images have provided the means for discovery, for description and for diagnosis. We consider a selection of different ways in which diseases have been located in this history: in the community, in the body, in the cell and on the image itself. These histories combine to give us new insights into images from the COVID-19 pandemic, the changing role of media in how we relate to disease, and what this can tell us more broadly about images as ‘subjective’ or ‘objective’ in relation to science.

## 2. Images with ‘style’

Medical illustration is now a thriving profession, with several courses, schools and associations focused on the training and development required. However, until at least the early twentieth century, such imagery was usually produced from the collaborative efforts of doctors and artists, or sometimes doctors acting as their own image makers [3].^2^ The history of illustrating both medicine, and science more broadly, involves three interweaving stories: improving knowledge of practitioners through direct study and observation, improved production techniques leading to changing styles and complexity of images, and developing imaging techniques allowing for ever more microscopic locating of disease in the human body. The result has often been a mutually beneficial cycle in which improved understanding has led to new types of image, which in turn influenced further research. Visualizing disease requires a combination of medical and visual discernment: the skill to be able to read an image and then turn that into another image which reflects what doctors or artists witness with their eyes [4].^3^

The modern history of scientific illustration starts in the sixteenth and seventeenth centuries with the publication of scientific texts that included printed illustrations. The invention of the printing press in the fifteenth century, and thus the move from manuscript to printed images allowed the development of what William Ivins has called ‘exactly repeatable pictorial statements' [5, pp. 1–2]. Pictures in the form of woodcuts, engravings and then etchings could become part of the argument of a text, cut into wood or metal by an artist and incorporated into the pages of a book usually as separate sheets. Books with images were popular from the sixteenth century, but often only with a pictorial frontispiece due to the costs associated with engraved plates. The invention of lithography in the late eighteenth century, in which images are printed from a stone or metal plate using the immiscibility of oil and water, allowed for the inclusion of cheaper and more plentiful images. However, this was not widely used in scientific texts until the 1820s. From the 1870s, photography further allowed for the reproduction of images produced as part of scientific activity, although other forms of imagery persisted alongside [6,7].

What, though, do we mean by an illustration? Brian Baigrie has discussed line drawing as ‘the simplest form of caricature in scientific illustration [which] lets the illustrator control exactly what the user sees’ [8, p. xx]. The reduction of an image to a series of lines simplifies the visual data for the viewer to the parts needed by the illustrator. It can play with levels of scale—both vast and minute—invisible to the human eye and simplify points of connection. The removal of shade and colour can direct what the viewer should look at but can also become unusable for someone not able to ‘read’ what they are seeing. The re-addition of colour and symbols to differentiate and label elements of the illustration, combined with a key, then encodes data within different parts of the picture. Every illustration requires the maker and user to share a set of visual conventions that allow the image to be read in the same way.

‘Style’ is thus as central a part of scientific images as it is the history of art, with changing ‘styles’ or conventions of representation shaping how images have been understood in different time periods [9, pp. 8–10]. We can identify innovations and patterns in scientific ‘style’ therefore as much as in artistic. Indeed, our widespread modern understanding of science as outside of this history of style and image has been traced by Martin Kemp to the development of ‘non-style’ as its own aesthetic for science in the nineteenth century. He describes this as greyness, ‘a technical mode of illustration in which the dry imparting of information is the sole conscious focus', and traces it particularly to the images produced for Henry Gray's *Anatomy, descriptive and surgical* first published in 1858, and thence an anatomical bible for generations of medical students [10, p. 70]. The consistent, plain and technical style of these illustrations ended what we more easily see as the ‘artistic’ medical illustration of previous centuries and spread into other branches of science.

## 3. Snow's Map

John Snow's (1813–1858) mortality map of the area of Victorian London surrounding Broad Street in Soho is an iconic scientific image, well-known across a number of disciplines, which shows the crucial significance of spatial plotting to understand and communicate the spread of disease. Snow was a physician in this area of London in the mid-nineteenth century, particularly interested in epidemiology and anaesthesia. He is best known for his work during the cholera epidemic of 1854 and for the specialist work as an anaesthetist that led to him administering chloroform to Queen Victoria at the births of two of her children.

**Figure 2.**
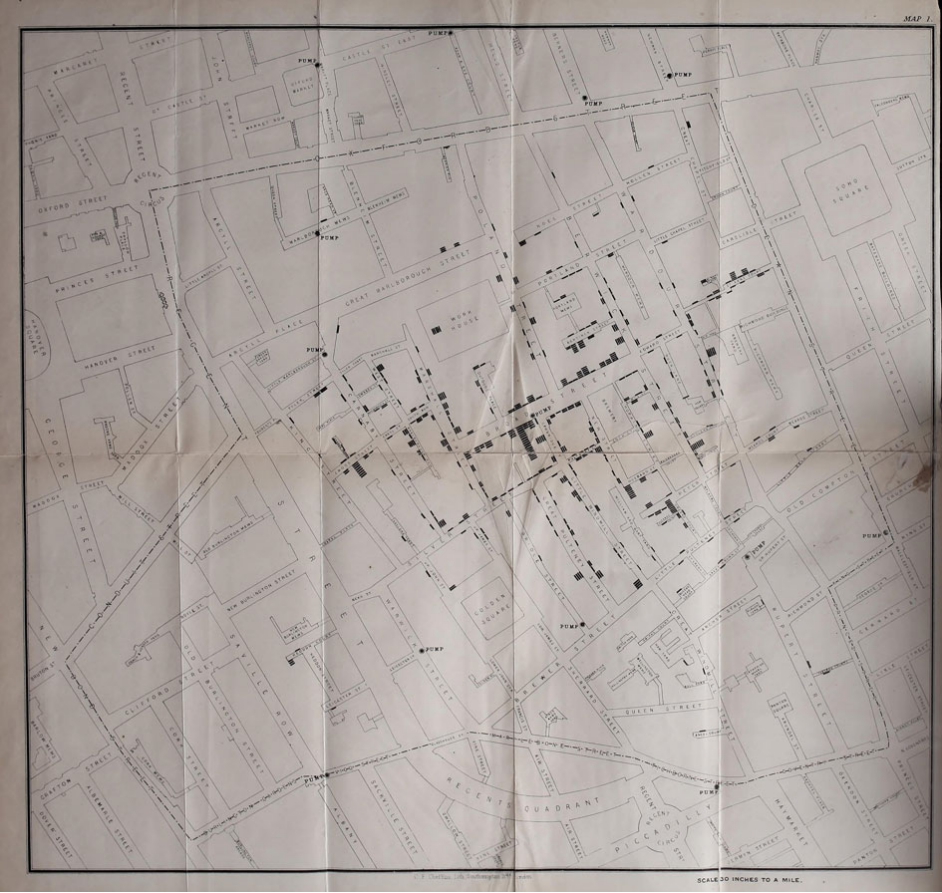
Map of the Broad Street pump from John Snow, On the mode of communication of cholera, 1855, Wellcome Collection.

Snow's work on cholera focused on two key findings, published in 1855 in the second edition of his *On the mode of communication of cholera* [11]. The first involved studying the water supply in districts of south London. By comparing deaths from cholera in areas supplied with water by the Lambeth Company as opposed to the Southwark and Vauxhall Company, he showed that residents were less likely to die if their water came from the former. He connected this to the fact that Lambeth had moved the source of their water upstream in the Thames, avoiding the sewage emptied in from London. The second focused on the water pump on Broad Street, in Soho. Snow used the list of deaths provided by the Registrar-General's office to work out the addresses of cholera victims. He then stood in the centre of Soho and noted their proximity to the Broad Street pump, also speaking to nearby families (figure 2).

Crucial to the argument of *On the mode of communication of cholera* is a map of the Broad Street area of London, on which the locations of various water pumps are marked. Snow used small black lines to mark the location of deaths or fatal attacks from cholera, showing simply but dramatically, the higher number of deaths near to the Broad Street pump. It clearly communicated his argument that the water from a specific pump was connected to a higher mortality rate and might therefore be the source of the disease. Medical geographers have disagreed over the precise role of the map in Snow's work: whether it was a tool produced as part of his methods or an illustration developed to communicate them [12,13]. Certainly, his accompanying text discussed observing the geography of the area to understand the significance of the pump, but the published map featured more deaths than would have been available to him as data at the time of his visit. Snow himself described the map as ‘a diagram of the topography of the outbreak’, and it seems reasonable that he developed a more compelling map for publication from a sketch used as part of his research [11, p. 45]. The illustration therefore works as both a tool and a message, using the recognizable form of the map to make a compelling case for the geography of disease spread. It does so at an understandable human scale of single lives located in London streets.

## 4. Nightingale's graph

**Figure 3.**
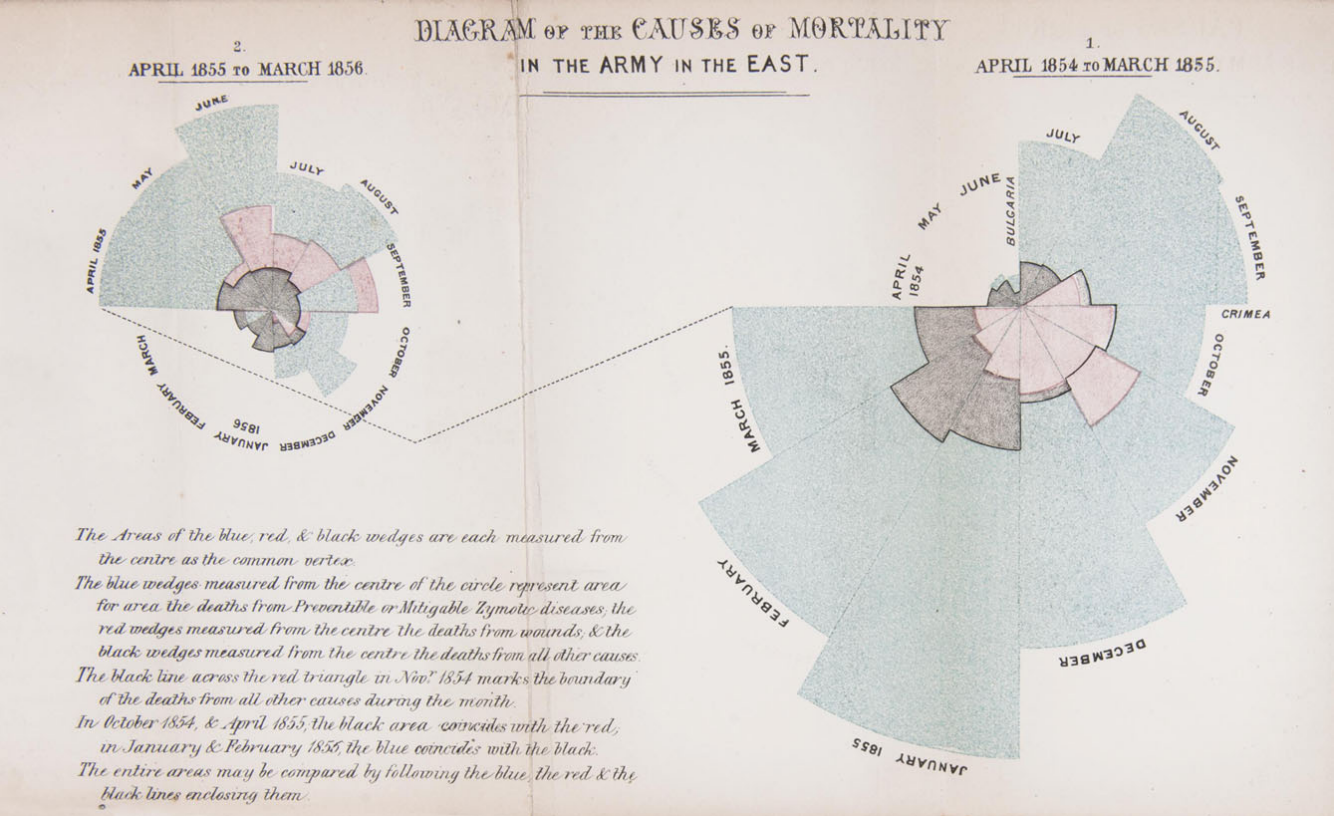
Plate from H. Martineau, England and her Soldiers, 1859. Science Museum Group © The Board of Trustees of the Science Museum.

In the same years in the middle of the nineteenth century, Florence Nightingale (1820–1910) was working as a nurse and administrator in the Crimea. She is best known from this period as ‘The Lady with a Lamp’ who tended wounded soldiers during the Crimean War and improved hospital sanitation. She is less well known for her pioneering work with statistics and their graphical representation, arguing for fundamental reform in how health data was gathered, both by the army and the medical profession more broadly. Nightingale worked closely with the Statistical Superintendent of the General Register office William Farr (1807–1883), a pioneering statistician and epidemiologist who made important use of visual representations in his work.^4^ Together they produced ground-breaking diagrams to show the impact of lack of sanitation in army hospitals. Nightingale was in the Crimea for under 2 years, 1854–1856, working to administer and oversee resources in the army hospitals, particularly at Scutari, as well as nursing directly. She saw first-hand how poor diet and sanitation led to the spread of disease, and thus to far greater deaths from preventable causes than from war wounds. On her return to London, she argued for a royal commission to be set up to consider the sanitary condition of the army, with the membership including Farr and three other statisticians. As a woman, she was not allowed to give evidence directly to the commission. Instead, she submitted written responses to questions, resulting in a subsequent report that included her own graphs drawn to visualize her arguments (figure 3).

*Notes on matters of the efficiency, health and the hospital administration of the British Army* was published in 1858. It included a persuasive circular graph, which Nightingale said she designed ‘to affect thro’ the Eyes what we fail to convey to the public through their word-proof ears' [14]. Her image is a polar area graph, which uses coloured sections and an extended radius to show the ‘causes of mortality in the army in the east’ between April 1854 and March 1856, representing thousands of deaths. Two graphs show 12 months each, with a segment for deaths within each calendar month: blue for preventable diseases, red for wounds and black for all other causes. The radius of each wedge is proportional to the contribution of each cause of death in that month. Nightingale shows very clearly how deaths from preventable disease hugely outweighed those from wounds.

Terminology for her new graph has been disputed. They are sometimes known as ‘rose’ diagrams or called ‘coxcombs’ or ‘wedges’ by taking terminology that Nightingale used for graphics published elsewhere, but M. Eileen Magnello has argued that ‘polar area graph’ is the most accurate name [15]. Nightingale's crucial innovation was in extending the radius of her graphs, making them more than a simple polar graph and adding drama to her data showing proportionate numbers of deaths, particularly with those from the preventable disease on the outside. Using the circle to show deaths in different months made the graph easier to read and understand, copying the passage of time familiar to her viewers from a clockface. While others had used similar graphs before, Nightingale's use of stylized line and colour choices made hers all the more striking, in many ways the prototype for all later uses. Her diagram was published in 1859 in Harriet Martineau's *England and her soldiers*, making her devastating representation of preventable deaths accessible to a wider public. It condensed the large datasets of deaths on a military scale into an image that made those numbers powerful.

## 5. Objective image-making

The nineteenth century was also the key period in which art and science became consciously separated into different disciplines, partly defined by their opposition to the other [9]. One of the drivers in this separation between art and science was the increasingly mechanized tools of vision, what Lorraine Daston and Peter Galison have described as ‘mechanical objectivity’^5^. For, alongside this history of illustration, image making has developed as a critical tool for exploring the roots of medical illness and disease itself. Microscopy had a long history as a tool of scientific observation dating back to the seventeenth century. In England, the iconic publication of *Micrographia* by the natural philosopher Robert Hooke (1635–1703) established the microscope as an essential tool for investigating the physical world beyond the scope of the unaided human eye [16]. However, in the nineteenth century, microscopy became an increasingly important tool for the medical and biological sciences when developments in achromatic and compound lenses allowed for the magnification of specimens with much greater clarity and lack of distortion [17]. Scientific societies, focused on the microscopical sciences flourished in this period, and a wide range of people, including doctors, naturalists and self-titled ‘amateur’ practitioners learnt how to prepare, examine and visualize soft tissue [18]. These societies published journals that were not only lavishly illustrated with a colour lithographic print but also accompanied by a matching slide preparation. Through preparing their own slides, subscribers combined the technical skill in slide preparation, the visual discernment to know what to look for through the lens of the microscope, and the craft of translating this through the hand and eye into a two-dimensional illustration. The boundary between images of art and science remained blurred.

By the start of the twentieth century, the separation between mechanically objective (i.e. scientific) and artistically subjective vision seemed to be becoming much more distinct. The development of the science of photography throughout this period played a significant role in this shift toward ‘mechanical objectivity’. By the 1870s, photochemical emulsions were able to capture details of the physical world that were well beyond the scope of human vision, allowing for the depiction of phenomena incredibly small, far away, or inside the human body. The imaging potentials open to scientists themselves were expanding, with the discovery of X-rays in 1895, the development of X-ray crystallography from 1912 and the deployment of three-dimensional models to understand atomic structures from the 1940s, to name just a few. Yet the role of images as tools for understanding and argument continued to prevail.

## 6. Schuster's X-ray

The year 1895 saw the invention of one of the most significant technological advances for the identification and diagnosis of illness: the X-ray. This photographic technology, developed by the German physicist Wilhelm Röntgen (1845–1923), has become a standard medical tool for identifying disease in the internal structures of the body. The X-ray became an essential tool in the twentieth century for medical practitioners not only to diagnose disease, but to be able to locate and track disease spread throughout the body. Oncologists, for example, rely on X-rays as the first tool of identifying cancer in patients, and they are used throughout cancer treatment to trace changes in cancerous growth. X-ray technology was also a foundational technological step towards the development of X-ray crystallography, the tool used by Rosalind Franklin (1920–1958) and Raymond Gosling (1926–2015) to create ‘photograph 51’ which was the critical data for the discovery of the structure of DNA [19].

**Figure 4.**
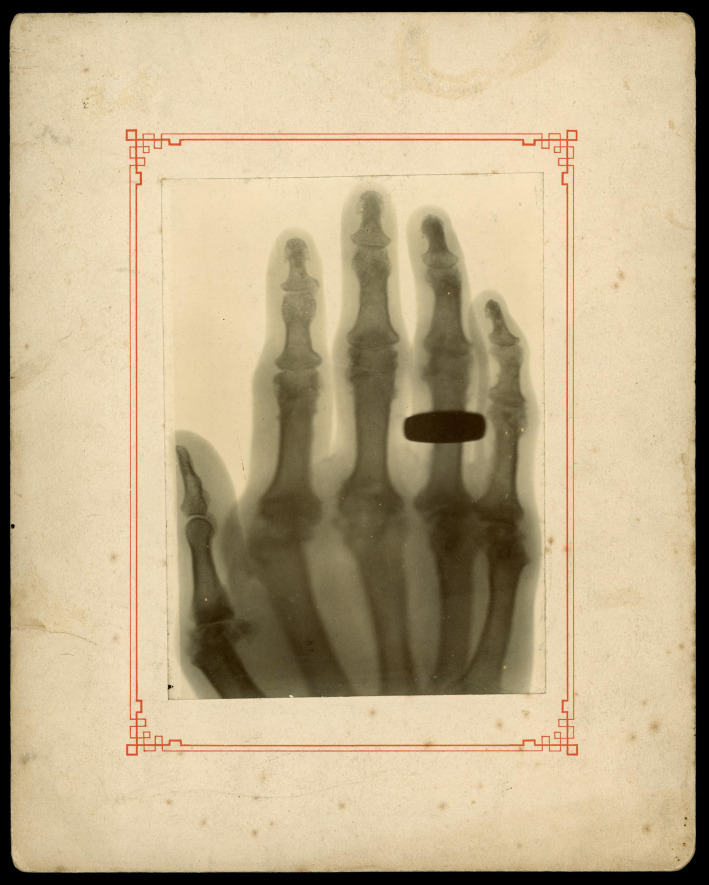
X-ray photograph of arthritic bones in a woman's hand by Prof. Arthur Schuster c.1896. SMG 1987-403/1. Science Museum Group © The Board of Trustees of the Science Museum.

Although X-rays tend to be championed as one of the most important tools of twentieth-century medicine, as Annie Jamieson has described, in the early decades of the century X-ray technology was not widely adopted by the medical community. There were alternative diagnostic tools for investigating the internal structures of the body, making X-rays novel experimental tools used to explore a new bodily aesthetic [20]. Following the practice established by Röntgen's famous X-ray image of his wife, Anna Bertha Ludwig's ‘hand with ring’, early experimenters with the X-ray explored similar subjects (figure 4).

Shortly after Röntgen's announcement of his new process, Arthur Schuster (1851–1934), a physicist at the University of Manchester, started his own experiments. Schuster created an X-ray image of a woman's hand who suffered from arthritis. From the outset, the subject of the image had medical implications. By studying the bone structure of this woman's hand, the X-ray could help identify the scale and progress of joint pain in this patient. Yet for a physicist with interests in the physics of light, particularly the two-decade-old science of spectroscopy and the recent innovations in radiography (or X-rays), this image became more than a diagnostic tool [21]. The fact that the patient is also wearing a ring in this image speaks to the desire to probe the limits of this new technology. What would become visible/invisible when a photographic plate was exposed to this specific wavelength of invisible light, known as an X-ray?

## 7. Caspar and Klug's comparison

Sixty-five years later, seeking to understand the visual form of the microscopic causes of many pandemic diseases—viruses—scientists turned to architectural structures alongside their own imaging techniques. In a seminal paper published in 1962, ‘Physical principles in the construction of regular viruses', Donald Caspar (born 1927) and Aaron Klug (1926–2018) used a wide range of imaging and illustration techniques to understand the shape of a simple virus [22, pp. 1–24].

**Figure 5.**
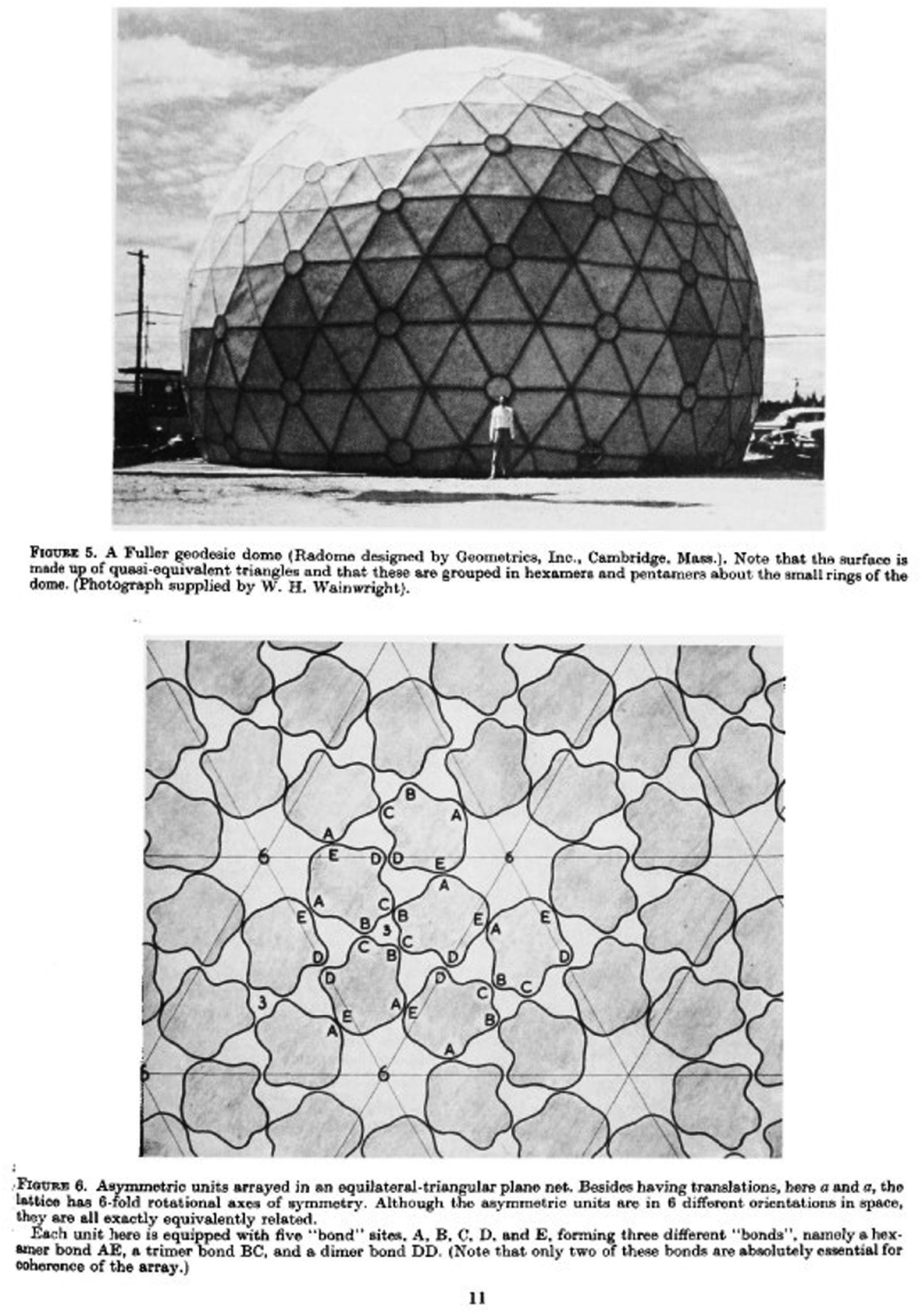
Page 11, Figures 5 and 6 from D. L. D. Caspar and A. Klug, ‘Physical principles in the construction of regular viruses’, Cold Spring Harbor Symposia on Quantitative Biology Volume XXVII, 1962 (Cold Spring Harbor Laboratory Press and W. H. Wainwright).

Their central question was what the most efficient structure for a virus shell would be, given that the small quantity of nucleic acid could only encode so much information: the structure would need to be formed from a number of identical protein molecules [23,24]. Francis Crick (1916–2004) and James Watson (born 1928) had hypothesized in 1956 that spherical viruses must therefore have cubic symmetry, with icosahedral symmetry the most likely as it allowed up to 60 identical units and thus a larger structure. Experimental evidence from X-ray crystallography and electron microscopy work by Caspar and Klug, the latter with collaborators John Finch (1930–2017) and Rosalind Franklin, also began to suggest that this was the case. Caspar started to use basic models, including ping-pong balls, to understand and communicate how such a structure might work [23]. The question was how to allow for more than 60 units (figure 5).

Through the interest of artist John McHale (1922–1978), Klug and Caspar became aware of the work of Buckminster Fuller (1895–1983). Still widely famous for his design of the geodesic dome, Fuller was a visionary inventor and architect. After Klug, Caspar and Fuller met in 1959, they shared notes, manuscripts and conversations, culminating in the scientists’ conclusion that virus shells are structured like microscopic geodesic domes. As they put it, they needed to ‘find a method of arranging more than 60 units on the surface of a sphere so that they are quasi-equivalently related … The solution we have found was, in fact, inspired by Buckminster Fuller in the construction of geodesic domes. Fuller has pioneered the development of a physically orientated geometry based on the principles of efficient design’ [22, p. 10]. Fuller's domes were constructed from repeating triangular faces, but not all the triangles were identical, a feature which allowed Klug and Caspar to develop the theory of ‘quasi-equivalence’: if the angle and length of bonds between protein units could vary slightly, like in the domes, virus structures with more than 60 units could be built. Their 1962 paper was densely illustrated, featuring an image of one of Fuller's domes among their sketches, models and X-ray diffraction patterns. Marshalling the image of the dome alongside their other (more expected) illustrations served to argue for the analogous structure of the dome and the virus, making their microscopic structures visible at a human scale.

## 8. Laboratory of Molecular Biology's vision

**Figure 6.**
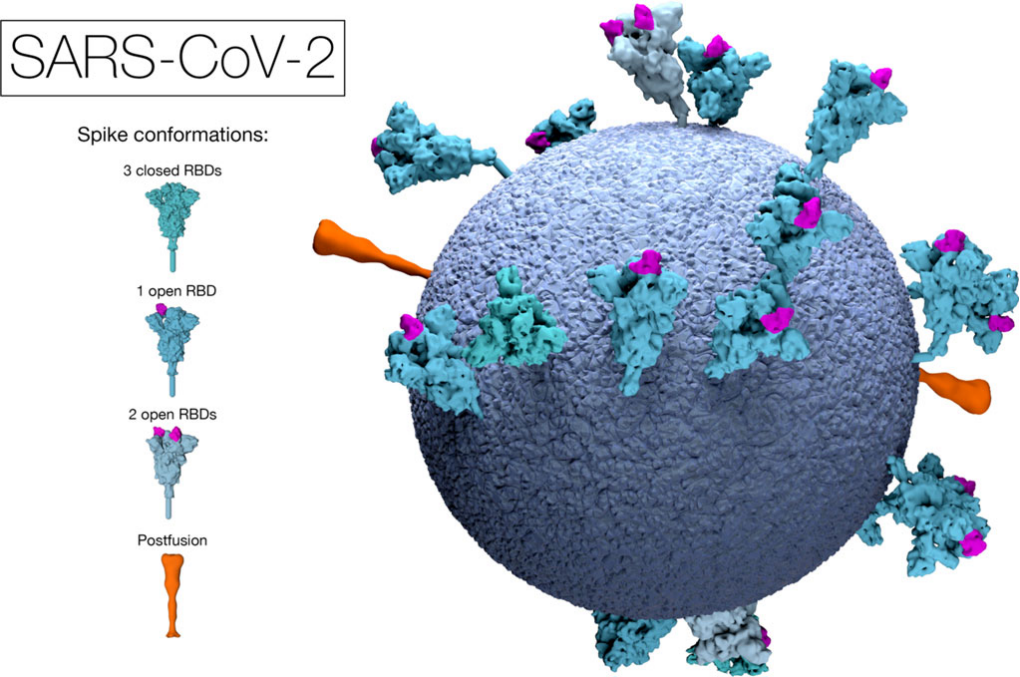
SARS-CoV-2 visualization from the Cambridge Laboratory of Molecular Biology. © MRC Laboratory of Molecular Biology.

In 2020, the role of imaging technologies came to the forefront in the challenge to identify the structure, and ultimately the cure to, the SARS-CoV-2 virus. The evolution of imaging tools throughout the twentieth century, particularly the electron scanning microscope, allowed scientists to investigate the molecular structure of inorganic substances. In the 1980s, the innovation of cryo-electron microscopy (cryo-em) gave molecular biologists the tools to map the structure of these substances, as it avoided the distortion created by the need to freeze specimens for use with an electron microscope. Cryo-em can therefore give an accurate image of what a virus looks like, which proved fundamental to the work of the scientific community in identifying SARS-CoV-2. While we might feel that we are very far from the ways in which science operated in the past—that the processes of science are now verifiable and free from human intervention—the way in which visual scientific data surrounding the coronavirus has been produced shows much remains the same (figure 6).

Take, for example, the work of the Cambridge University Laboratory of Molecular Biology (LMB) to help identify the structure of SARS-CoV-2. Throughout 2020, John Briggs's laboratory team studied the protein spikes of SARS-CoV-2 using cryo-em [25]. They produced a model of the virus, which they argued identified the ‘authentic’ position of the protein spikes on the virus.^6^ The positions of the spikes were critical for identifying how the virus was able to replicate and also demonstrate the ‘crown’ of spikes which gave the virus its name. This image was crucially different from the better-known image of the coronavirus produced by the Centers for Disease Control and Prevention (CDC) in the USA, which was used widely within the media when talking about COVID-19.^7^ While the CDC image was an evocative representation of the virus, it was not meant to represent an ‘authentic’ depiction. It became, using Ivin's phrase, an ‘exactly repeatable pictorial statement’ useful for public understanding of the virus, but different to the LMB image rooted in data.

In order to create the model for SARS-CoV-2, the LMB team worked in collaboration. Using the data from cryo-em tomography of virus samples, the team was able to locate the specific position of the spikes in three dimensions on the virus. This required a combination of static two-dimensional images that were stacked together to create a three-dimensional volume of the virus. Working with their in-house data visualization team, the set of images from different viruses was then processed through modelling software that helped turn the image into visually appealing yet scientifically accurate images. At this stage key, aesthetic decisions were made, such as which colour to apply to each section of the virus, or from which vantage point to position the model for public consumption. The end result was an image which combines mechanical objectivity and aesthetic choice, continuing the long history of collaboration between image makers and medical practitioners to locate disease by bringing together scientific observation and imaging techniques.

## 9. Conclusion

**Figure 7.**
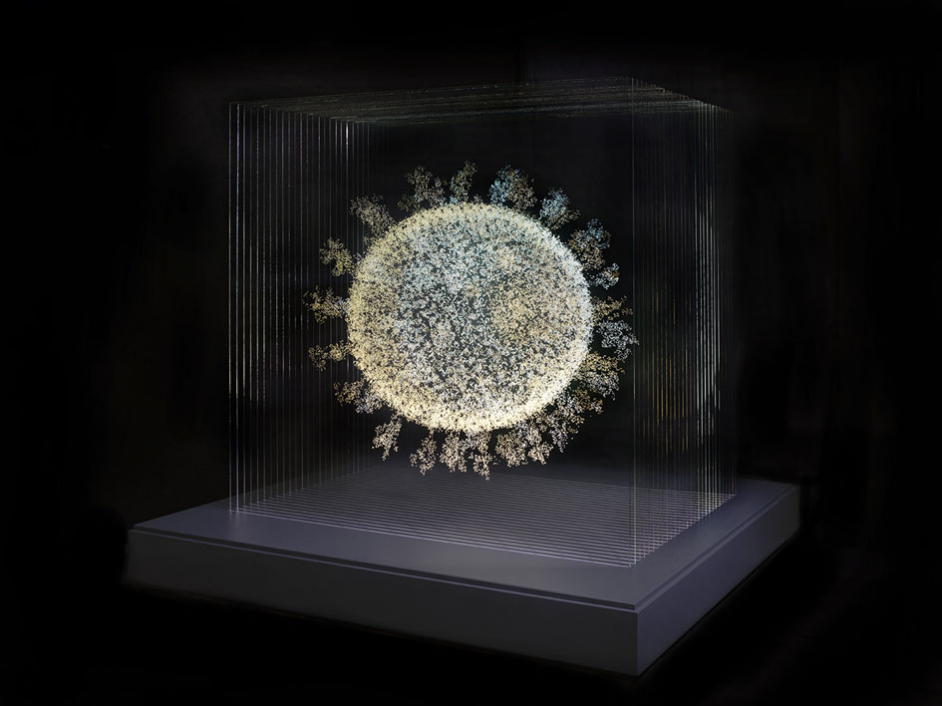
Angela Palmer, 2020: the Sphere that Changed the World (2020). Photo: Andrew Smart, A. C. Cooper.

One of the more striking visual results of the COVID-19 pandemic has been the power of the image of the coronavirus. From a medical illustration, produced by experts at professional institutions like the CDC or LMB, the crowned circle has entered popular visual culture appearing in cartoons, artworks and even as an emoji. No previous virus or disease has gained such visual currency for the image of its source, an image rooted in processes of scientific imaging and illustration. British artist Angela Palmer has produced a striking glass sculpture of the coronavirus. Her work combines scientific data from the genomic map of the Wuhan virus with medical imaging techniques developed from MRI scanning. She actively combines cutting-edge work in scientific analysis and imaging with the long history of artists illustrating disease (figure 7).

Images are ways to identify, locate, describe and replicate information about illness. The history of how images have been used by science and medicine has been fundamentally linked to the tools of their creation and replication. Innovations such as new ways of mapping disease or the application of X-ray or cryo-em technology have allowed us to investigate disease in new ways. Yet the making of these images has not been value free; they are laden with meaning: meaning taken from data, from our tools of observation, from the contexts of their use and from aesthetic choice. The global effort to solve the coronavirus pandemic has been an unprecedented achievement of scientific, medical and social collaboration. While this achievement has many contributors, the production and use of image technologies, as we have demonstrated in this article, has played a significant role.

## Endnotes

1 See examples from ‘Embedding the Human Disease Network’ at http://snap.stanford.edu/deepnetbio-ismb/ipynb/Human+Disease+Network.html.

2 A key moment in the history of the profession is the founding of the Johns Hopkins School of Medical Illustration by German artist Max Brödel in 1911. See [3].

3 Describing the effects of disease on the human body has a long history, as Richard Barnett describes in his book *The Sick Rose.* Based on images from the Wellcome Collection, Barnett traces the history of western illustration practices of depicting the diseased human body, from Vesalius onwards. Image making, for Barnett, is a tool both for documenting and for communicating with other doctors about how different diseases present on the human body. See [4].

4 Farr also provided data to Snow.

5 Daston and Galison, *Objectivity*.

6 https://www2.mrc-lmb.cam.ac.uk/lmb-joins-the-fight-against-covid-19/.

7 https://phil.cdc.gov/Details.aspx?pid=23311.

## Data accessibility

This article has no additional data.
